# Fabrication of a controlled-release delivery system for relieving sciatica nerve pain using an ultrasound-responsive microcapsule

**DOI:** 10.3389/fbioe.2022.1072205

**Published:** 2022-11-24

**Authors:** Xiong Xu, Shuai Chang, Xiaoyi Zhang, Taotao Hou, Hui Yao, Shusheng Zhang, Yuqi Zhu, Xu Cui, Xing Wang

**Affiliations:** ^1^ Department of Orthopaedics, The 8th Medical Center of PLA General Hospital, Beijing, China; ^2^ Department of Graduate, Hebei North University, Zhangjiakou, China; ^3^ Beijing National Laboratory for Molecular Sciences, Institute of Chemistry, Chinese Academy of Sciences, Beijing, China; ^4^ Orthopedics Department, Peking University Third Hospital, Beijing, China; ^5^ Department of Orthopedics, Eye Hospital, China Academy of Chinese Medical Sciences, Beijing, China; ^6^ ShenYang Tiantai Remote Medical Tech Development Co., Ltd., Shenyang, China

**Keywords:** microcapsule, lidocaine, ultrasound responsive, sciatica relief, on-demand administration

## Abstract

Lidocaine, a potent local anesthetic, is clinically used in nerve block and pain management. However, due to its short half-life, repeated administration is required. For this reason, here we designed and prepared a lidocaine-encapsulated polylactic acid-glycolic acid (Lidocaine@PLGA) microcapsule with ultrasound responsiveness to relieve the sciatica nerve pain. With a premixed membrane emulsification strategy, the fabricated lidocaine-embedded microcapsules possessed uniform particle size, good stability, injectability, and long-term sustained release both *in vitro* and *in vivo*. More importantly, Lidocaine@PLGA microcapsules had the function of ultrasonic responsive release, which made the drug release controllable with the effect of on-off administration. Our research showed that using ultrasound as a trigger switch could promote the rapid release of lidocaine from the microcapsules, achieving the dual effects of long-term sustained release and short-term ultrasound-triggered rapid release, which can enable the application of ultrasound-responsive Lidocaine@PLGA microcapsules to nerve root block and postoperative pain relief.

## Introduction

Lidocaine was first synthesized in the 1940s and was initially used as a local anesthetic and antiarrhythmic treatment (Hermanns et al., 2019). In the 1950s, as research on the molecular mechanism of lidocaine deepened, it was found that lidocaine can not only block sodium ion channels (Gawali et al., 2015) but also has critical effects on hyperpolarization-activated cyclic nucleotide-gated (HCN) channels. A certain inhibitory effect, which changes the conduction of action potentials, had analgesic properties (Zhou et al., 2015; De Cassai et al., 2021; Foo et al., 2021; Lee and Schraag, 2022) and played an important role in pain management (Dunn and Durieux, 2017; Soto et al., 2018). However, due to its poor water solubility and short half-life, the drug effects only last for 2 h, and thus it often requires the repeated administration to obtain satisfactory results to satisfy clinical application (Liu and Lv, 2014). In general, encapsulation of drugs into polymeric carriers has attracted emerging interests in recent years. Scientific teams have designed and developed sustained-release materials including microcapsules, nanoparticles, hydrogels, liposomes, and other sustained-release materials as drug carriers (Ma, 2014; Svirskis et al., 2016; Nagasaki et al., 2017; Butreddy et al., 2021; Escareño et al., 2021; Li et al., 2021; Zeng et al., 2021), and the embedded drugs in the sustained-release materials can provide long-term slow release under mild conditions (Sharma et al., 2018). However, due to differences in degree of pain and the pain tolerance, individualized treatment and on-demand administration are required in practical clinical applications, as the long-term sustained-release function alone also does not meet clinical needs (Suraphan et al., 2020).

Ultrasound-responsive polymeric materials had attracted significant attention for several decades. Compared to other traditional stimulate respondence, such as UV stimulus, thermal stimulus, and pH stimulus, the ultrasound-responsive materials were more applicable because of their efficient drug delivery and targeted treatment *via* non-invasive means. Commonly used ultrasound-responsive biomaterials mainly included micro/nano bubbles/droplets, polymeric micelles, prodrugs, hydrogels, and nanogels (Marin et al., 2001; Rapoport et al., 2011; Huebsch et al., 2014; Hernandez et al., 2017; Shende et al., 2018; Tang et al., 2018; Shende and Jain, 2019; Wei et al., 2020). Various drugs or bioactive molecules can therefore be delivered using these ultrasound-responsive delivery vehicles for a variety of disease therapies (Doukas and Kollias, 2004; Chung et al., 2008; Aryal et al., 2014; Yin et al., 2014; Yamaguchi et al., 2019; Wang et al., 2022).

**FIGURE 1:**
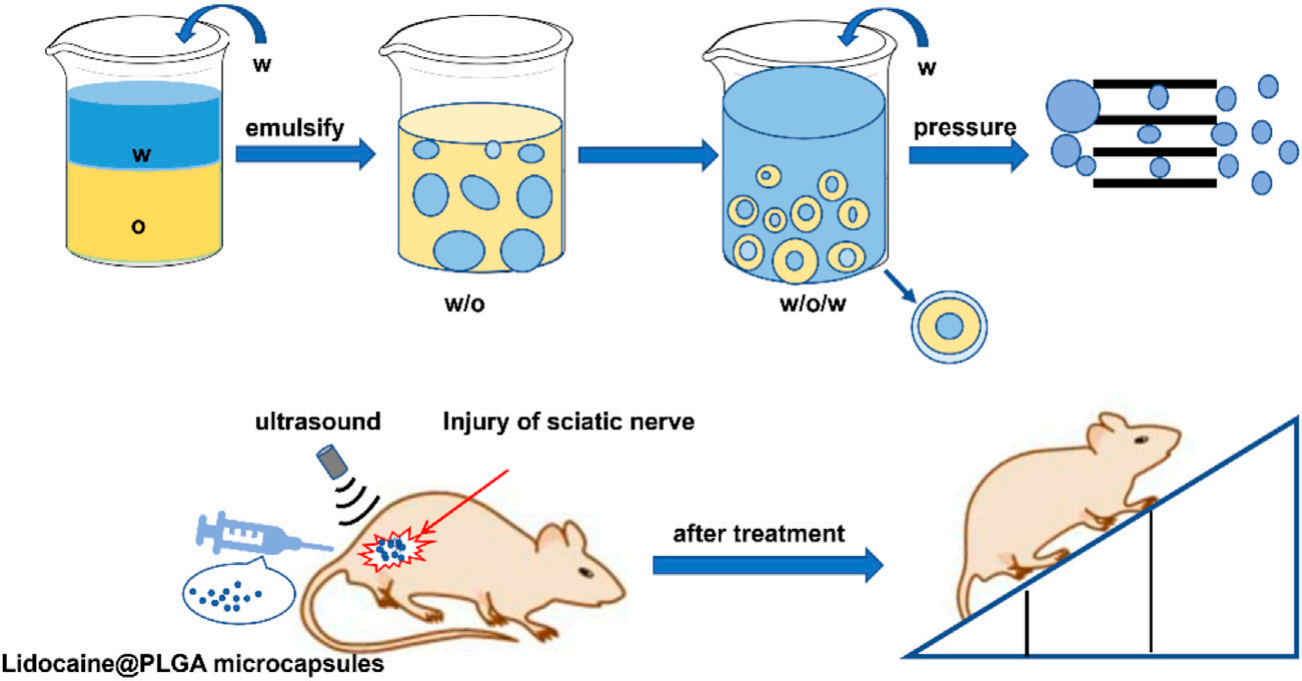
Schematic diagram of the preparation of Lidocaine@PLGA microcapsules *via* the PME method and their application in the rat sciatic nerve injury model.

In this work, we successfully prepared ultrasonic-responsive Lidocaine@PLGA microcapsule formulations by encapsulating insoluble lidocaine drugs inside the microcapsules through premix membrane emulsion (PME) combined with the water in oil in water (W/O/W) double emulsion method (Figure 1). Rational design made the Lidocaine@PLGA microcapsules uniform in particle size, well dispersible, and injectable. In addition, as a non-invasive external mechanical energy, ultrasound can controllably adjust the drug release of drug-loaded microcapsules to achieve on-demand drug delivery (Kost et al., 1989; Sirsi and Borden, 2014; Wallace and Wrenn, 2015; Gao et al., 2018; Chandan et al., 2020; Ben Daya et al., 2021; Yuan et al., 2021; Fan et al., 2022). The polymer PLGA is an important macromolecular organic compound with good biodegradability and biocompatibility, which is widely used in the field of biomedicine. Thanks to their unique encapsulation and globularity, some drugs, proteins, and vaccines can be encapsulated for long-term sustained slow release(Cappellano et al., 2019; Ospina-Villa et al., 2019; Rocha et al., 2022). *In vitro* and *in vivo* experiments confirmed that microcapsules with ultrasound responsiveness can switch drug delivery to become continuously released for more than 10 days, achieving dual effects of long-term sustained release and short-term ultrasound-triggered rapid release, achieving a novel on-demand drug delivery and thereby meeting a range of clinical requirements. The combination of polymer PLGA and ultrasound is a new discovery in medical materials, and it meets the clinical needs of long-term sustained release and short-term rapid release under ultrasonic intervention, which is helpful for deeper exploration and wide application in the field of biomedicine.

## Materials and methods

### Materials

PLGA (weight: 10,500; lactide/glycolide ratio, 75/25) was purchased from Yuanbohuike Institute of Biotechnology (Beijing, China). Lidocaine was purchased from Sarn Chemical Technology Co., Ltd. (Shanghai, China). Polyvinyl alcohol (PVA) was purchased from Shanghai Aladdin Biochemical Co., Ltd. (Shanghai, China). Dichloromethane was purchased from Concord Technology Co., Ltd. (Tianjin, China). The specification of the dialysis membrane purchased from Solarbio Technology Co., Ltd. (Beijing, China). Rapid membrane emulsification equipment (FMEM-500M) and Shirasu Porous Glass (SPG) membrane were purchased from National Engineering Research Center for Biotechnology. The SPG membranes were annulus cylinders with pore sizes of 7.0 μm. A hand-held ultrasound (Model TT-CC-X convex array) apparatus was purchased from Shenyang Tiantai Telemedicine Technology Development Co., Ltd. (Shenyang, China). A cell-crushing apparatus was purchased from Shanghai Xinzhi Biotechnology Research Institute (Shanghai China). An ultrasonic water bath apparatus was purchased from Kunshan Ultrasonic Instruments Co., Ltd. (Kunshan China). A high-speed centrifuge was purchased from Anhui Jiawen Instrument Equipment Co., Ltd. (Anhui China).

### Measurements

The morphology of the microcapsule was observed with scanning electron microscopy (SEM; JSM-6700F, JEOL, Japan) at an accelerating voltage of 5 kV. An aqueous solution of drug-loaded microcapsule particles was adhered to a metal circular table using conductive glue and vacuum-coated with a thin layer of platinum using a sputter coater (EM SCD 500, Leica, GER). Fourier transform infrared (FT-IR) spectra were recorded using the potassium bromide pellet method and an infrared spectrometer (Tensor 27, Bruker, GER). The microcapsule size was measured with dynamic light scattering (DLS, Nano-ZS90, Malvern, U.K.).

### Preparation of Lidocaine@PLGA microcapsules

Lidocaine@PLGA microcapsules were prepared using a water-in-oil-in-water (W/O/W) membrane emulsification method. First measure 3 ml of deionized water as the inner water phase. An appropriate amount of 1% PVA aqueous solution was prepared as the outer water phase, and 400 mg of PLGA and 400 mg of lidocaine were dissolved in 8 ml of dichloromethane as the oil phase. The inner water phase was added to the oil phase, and emulsified by ultrasonic (power 200 W) with an ultrasonic cell disruptor to obtain a water-in-oil primary emulsion. The primary emulsion was then poured into 200 g of the outer aqueous phase to form a water-in-oil-in-water pre-reconstituted emulsion. The pre-recombination emulsion was quickly poured into the membrane emulsification device, and the re-emulsion emulsion was pressed through a membrane tube with a pore size of 7.0 μm with a nitrogen pressure of 0.07 MPa, repeated three times until a re-emulsion emulsion with uniform particle size was obtained. After stirring overnight at room temperature to allow the solution to completely evaporate, the solidified microspheres were collected by centrifugation, washed three times with distilled water, and freeze-dried in a liquid nitrogen freeze dryer for 24 h to obtain the targeted Lidocaine@PLGA microcapsules.

### Determination of drug loading

A certain weight of Lidocaine@PLGA microcapsules was added into an appropriate amount of dichloromethane to dissolve. Then hydrochloric acid solution was extracted three times, mixed, and diluted into a bottle, and the absorbance was measured at 262.5 nm by UV spectrophotometry. The amount of lidocaine in the solution was calculated as follows.

$$Drug\;loading\;\left(\%\right)=\frac{lidocaine\;amount\;\left(mg\right)}{Lidocaine@PLGA\;microcapsules\left(mg\right)}*100\%$$ (1)

### *In vitro* drug release

**FIGURE 2:**
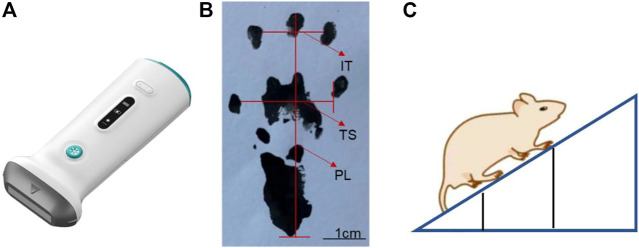
Schematic diagram of **(A)** handheld ultrasound instrument, **(B)** sciatic nerve index detection, and **(C)** oblique plate experiment.

The suspension of physiological saline and drug-loaded microcapsules was put into a dialysis tube (molecular weight cut-off: 3500), and the lidocaine release behavior of Lidocaine@PLGA microcapsules in the dialysis tube was monitored at 40 r/min in a constant temperature shaker at 37°C. To verify the ultrasound-responsive behavior of drug-loaded microcapsules, we used and compared three instruments: a handheld ultrasound(Figure 2A), a water bath ultrasound, and a cell disruptor. The suspension of normal saline and drug-loaded microcapsules was put into a dialysis tube (molecular weight cut-off: 3500) and exposed to ultrasound instruments for the predetermined duration time. The control group was placed in a thermostatic shaker for 40 r/min without any intervention. At various time intervals, 5 ml solution outside the dialysis membrane was withdrawn and replaced with a freshly mixed solution. The absorbance was brought into the standard curve equation by UV spectrophotometry to calculate the cumulative release percentage, as follows.

$$Cumulative\;release\;percentage\;\left(\%\right)=\frac{V_{0}*C_{n}+V*\overset{n-1}{\underset{i=1}{\sum }}C}{M*D}*100\%$$ (2)

V_0:_ Volume of release medium, mL; C_n_: Concentration of lidocaine hydrochloride at the *n*th sampling point, mg/mL; V: volume per sample, mL; M: The total mass of the microspheres in the delivery medium, mg; D: Drug loading of microspheres in release medium.

### *In vitro* cytotoxicity assay

L929 cells were seeded in 96-well plates (30,000 cells per well) and cultured at 37°C in a 5% CO_2_ for 24 h. Then, the prepared lidocaine microcapsules and blank microcapsules in different aqueous solutions were added to the orifice plate at 10-, 50-, 100-, 150-, and 200-fold dilution concentrations, and the unmedicated group was used as a negative control. After 24 h incubation, cell viability was calculated using cell Counting Kit-8 (CCK-8, Dojindo, Kumamoto, Japan) according to the manufacturer’s instructions. Absorbance was read at a wavelength of 450 nm using a microplate reader (Varioskan Flash; Thermo Fisher Scientific, Waltham, MA, United States).

### Establishment of animal models

In all, 12 healthy male SD rats aged 6–8 weeks with a body weight between 250 and 300 g were randomly divided into sham surgery group, lidocaine microcapsules group, and lidocaine microcapsules ultrasound group, with 4 in each group. To each, 0.1 ml/100 g of 3% pentobarbital intraperitoneal anesthesia was given; after the anesthesia is satisfactory, the skin was prepared on the back of the left lower extremity. After disinfection, an incision is performed of the skin and the subcutaneous fascia, and hemostatic forceps were used to separate the biceps femoral muscle and the hemimetic muscle until the coarse sciatic nerve was visible; blunt separation and ligation were performed with 4-0 suture at the front 2 mm of the trigeminal branch, in a ligation range is 5 mm and a ligation spacing is 1 mm, with the degree of ligation tightness subject to the twitching of the calf muscles. Physiological saline irrigation and hemostasis of the wound were performed after the operation was completed. The muscles, skin, and surgery area were disinfected in turn with alcohol. Rats in the sham surgery group were only free from the sciatic nerve and were not ligated. The experimental procedure ensured that all rat muscles were sutured with one stitch two skin sutures, and the entire surgical process was completed by the same operator.

### Determination of sciatic nerve function index

Ink was dripped on the soles of the feet on both sides of the SD rats, and they were placed in the starting position on the paper runway. The two sides of the runway were covered with cardboard pieces to prompt the rats to run to the end of the runway. The bottom of the runway was covered with white pieces of paper for easy recording of the rat footprints. The length of the runway was optimal for collection of the footprints of the 10 pairs of rats, and the width of the runway was just sufficient to accommodate the rats. The distances of the parts of the rat’s footprint were measured at different time points (Figure 2B), and the sciatic nerve function index of rats was evaluated as follows.

SFI=109.5(ETS−NTS)/NTS−38.3(EPL−NPL)/NPL+13.3(EIT−NIT)/NIT−8.8 (3)

SFI = 0 indicates normal sciatic nerve function,

SFI = -100 represents complete sciatic nerve injury. Here, experimental foot (E), normal foot (N), print length (PL), toe spread (TS), and intermediate toe spread (IT) are defined.

### Inclined plate experiment

The rats were placed on the rectangular cube floor in turn, and after the rat adapted to the surrounding environment, they were close to the rectangular cube head side, and the body axis of the rat was kept perpendicular to the rectangular cube head side. This side was mounted with a lifter, so that it could be slowly raised, and the angle between the three-dimensional box bottom plate and the horizontal surface gradually increased. When the rat was had difficulty remaining attached to the three-dimensional box bottom plate for 5 s and began to slide down, the angle between the three-dimensional box floor and the horizontal surface was recorded (Figure 2C).

## Results and discussion

### Preparation and characterization of Lidocaine@PLGA microcapsule

**FIGURE 3:**
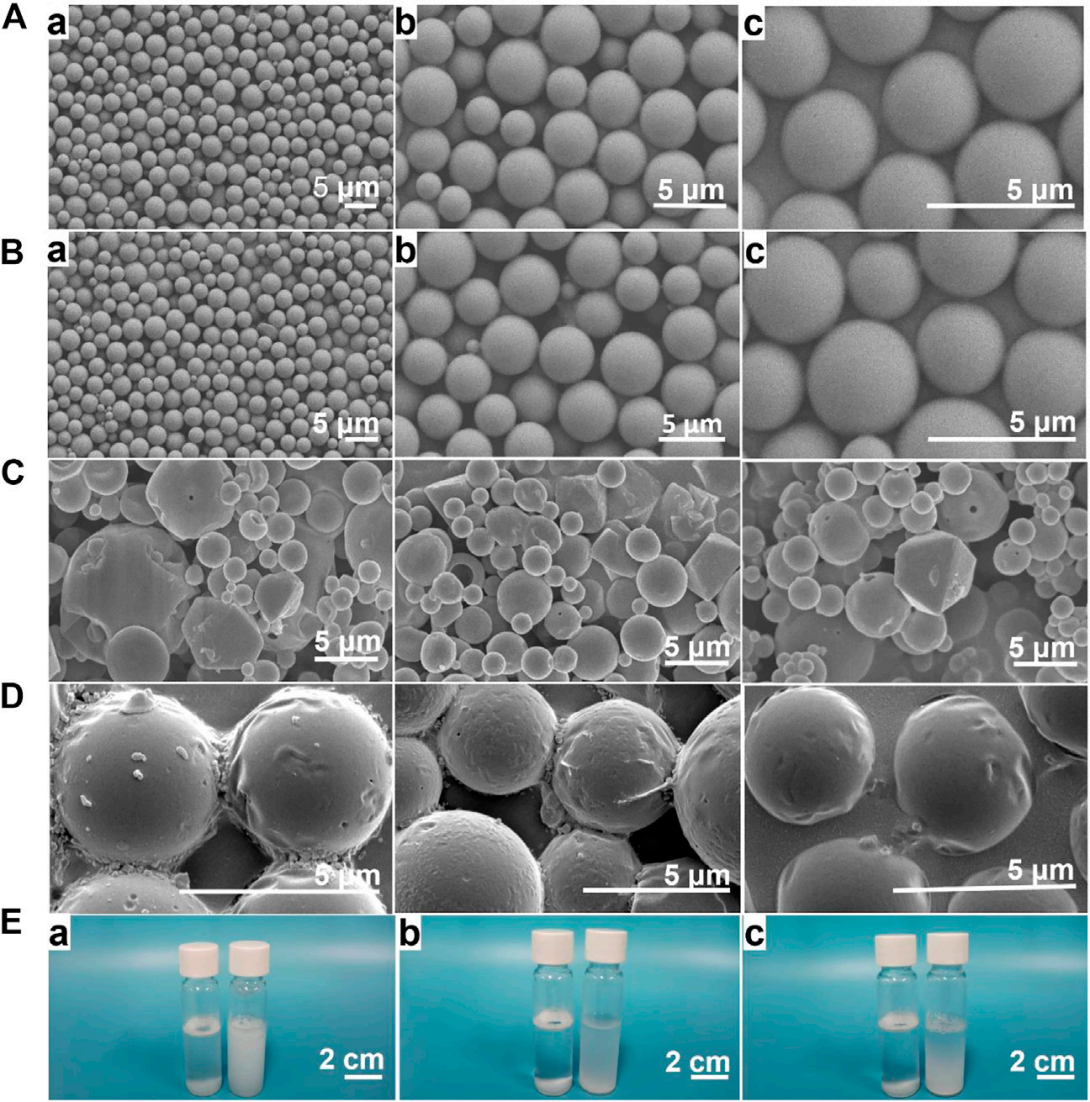
**(A)** PLGA microcapsule prepared with premixed membrane emulsification method. Lidocaine@PLGA microcapsule prepared with **(B)** and without **(C)** premixed membrane emulsification method by electron microscope at (a) 1000x, (b) 3000x, or (c) 5000x. **(D)** The morphology change of Lidocaine@PLGA microcapsule after ultrasound process. **(E)** Optical photographs of 3 mg/ml of Lidocaine@PLGA microcapsule with (right) and without (left) premixed membrane emulsification method in water after standing for (a) 0 h, (b) 6 h, and (c) 12 h.

Scanning electron microscopy (SEM) showed that PLGA microcapsules and Lidocaine@PLGA microcapsules were spherical, with a regular shape and smooth surface (Figures 3A,3B), with no significant difference. By contrast, the prepared Lidocaine@PLGA microcapsules without premixed membrane emulsification method exhibited heterogeneous, unsystematic, and even disorderly morphologies and rough surface (Figure 3C). Then, we further observed the effect of the ultrasound on the microcapsules after a hiatus of 2 weeks and found that the surface was gradually changed from the smooth to the rough along the occurrence of some fold structures in Figure 3D, which indicated ultrasound-responsive drug release behavior from the Lidocaine@PLGA microcapsule. The obtained microcapsules with uniform particle size of ca. 3 μm were uniformly dispersed, and good suspension stability was seen under different magnifications. Relative to the traditional solvent evaporation method, the Lidocaine@PLGA microcapsule prepared by the membrane emulsification method can be evenly distributed in solutions even after standing for 12 h, which is beneficial for clinical application (Figure 3E).

**FIGURE 4:**
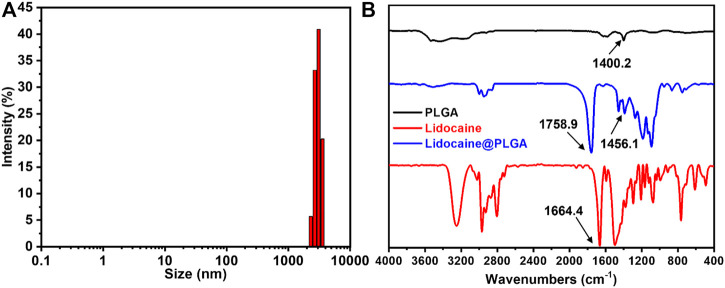
**(A)** DLS profile of the Lidocaine@PLGA microcapsule. **(B)** IR spectra of the drug, PLGA polymer, and the Lidocaine@PLGA microcapsule.

The diameter of the Lidocaine@PLGA microcapsule was further measured using the DLS result, which exhibited a similar size to that seen in Figure 4A. The infrared spectrum results suggest the appearance of certain typical characteristic peaks of PLGA polymer and lidocaine. More importantly, some characteristic shift peaks were seen from 1400.2 cm^−1^ to 1456.1 cm^−1^ and from 1664.4 cm^−1^ to 1758.9 cm^−1^ (Figure 4B), which indicated a physical interaction between the two substances and lidocaine within the lidocaine microcapsules, powerfully revealing that the lidocaine was successfully encapsulated into the microcapsules, rather than being the result of a simple physical mixing.

**FIGURE 5:**
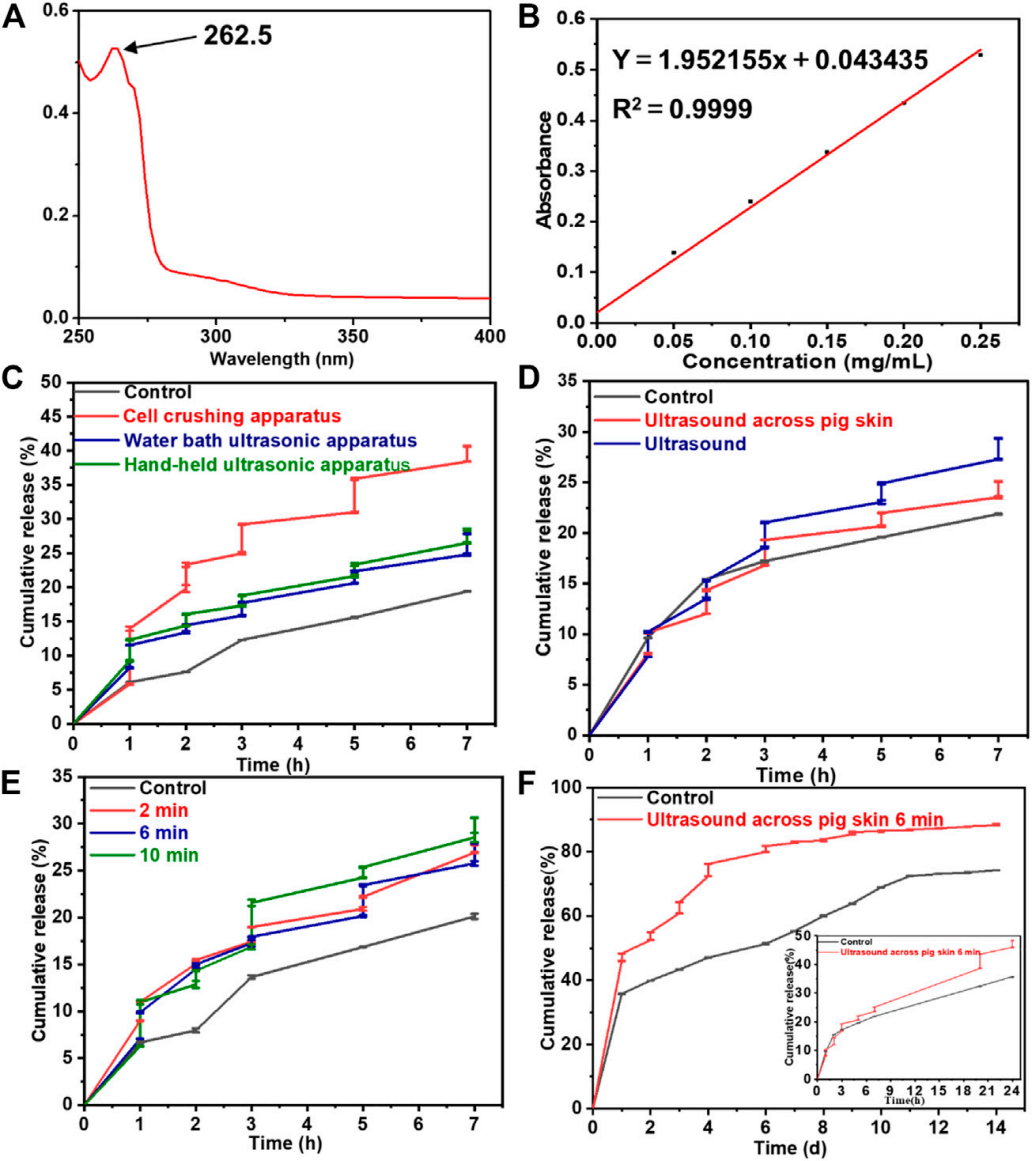
**(A)** The UV-scanning spectrum and **(B)** the standard curve of lidocaine. The drug release behaviors from Lidocaine@PLGA microcapsules with various **(C)** ultrasonic equipment, **(D)** ultrasound across pig skin, **(E)** ultrasonic duration time, and **(F)** cumulative release with each specific ultrasonic duration time of 6 min.

The drug loading ratio of the Lidocaine@PLGA microcapsule was calculated as 15.5%. We investigated the ultrasound-responsive drug release of Lidocaine@PLGA microcapsules with a handheld ultrasound, a water bath ultrasound, and a cell rupture device, and we compared them with a blank control group. The UV-scanning spectrum and standard curve of Lidocaine are displayed in Figures 5A,5B, exhibiting the absorbance was at 262.5 nm by UV spectrophotometry. As shown in Figure 5C, Lidocaine@PLGA microcapsules were performed for continuous ultrasound of 6 min at 1, 2, 3, 5, and 7 h, respectively. After 7 h, the cumulative drug release rates of the blank control group, the handheld ultrasound group, the water bath ultrasound group, and the cell crushing apparatus group were calculated to 19.4%, 28.4%, 27.8%, and 40.7%, respectively. Moreover, the step-like release curve appears during the ultrasound stimulation process, indicating that the Lidocaine@PLGA microcapsules had good ultrasonic stimulation response according to these three ultrasound instruments. However, considering the facile manipulation, simple daily application, and ultrasound-responsive effects, we selected the feasible handheld ultrasound form Shenyang Tiantai Telemedicine Technology Development Co., Ltd. for the following ultrasound-responsive experiments, indicating that this kind of novel handheld ultrasonic instrument could have a broader application prospect in the clinic.

To verify whether the Lidocaine@PLGA microcapsules were responsive to handheld ultrasound when applied *in vivo* with skin barrier, we simulated a skin barrier by covering the surface of the handheld ultrasonic probe with a fresh pig skin. The results in Figure 5D showed that after the cumulative release of drugs in the pigskin ultrasound group after 7 h, the direct ultrasound group and the blank control group showed values of 25.1%, 29.3%, and 21.9%, respectively, which confirmed the simulated skin barrier conditions Lidocaine@PLGA microcapsules could be responsive to handheld ultrasound. To further increase the duration time of ultrasound on the drug release of Lidocaine@PLGA microcapsules, we covered the surface of the ultrasound probe with a layer of fresh pig skin and performed continuous ultrasound at the corresponding time points for 2 min, 6 min, and 10 min, respectively. The results in Figure 5E showed that the longer the period that the ultrasound time could cause the more obvious the stimulation response of the Lidocaine@PLGA microcapsules. Because long ultrasound times can damage the surrounding tissues and it was difficult to ensure a sufficiently long ultrasound time for a single treatment during clinical application, we selected 6 min as the optimal ultrasound duration time. Finally, we studied the long-term effects of handheld ultrasonography on Lidocaine@PLGA microcapsules under simulated skin barrier conditions, and the results showed that the ultrasound group and the control group had a burst release period in the first 20 h, with a cumulative release rate of 43.6% and 32.3%, respectively. Then these two curves gradually entered a slow-release period with gentle cumulative release behaviors. Figure 5F showed that cumulative release curve of ultrasound group at day 9 tended to flat with a high cumulative release rate of 86.2%, while the control group lasted until 11 days with a cumulative release rate of 72.4%. These *in vitro* experiments verified that Lidocaine@PLGA microcapsules with intermittent handheld ultrasound of 6 min under simulated skin conditions were still efficient for performing the ultrasound-responsive drug release, which could meet the dual needs of clinical long-term sustained release and/or temporary “on-off” control release.

### Cell viability and proliferation

**FIGURE 6:**
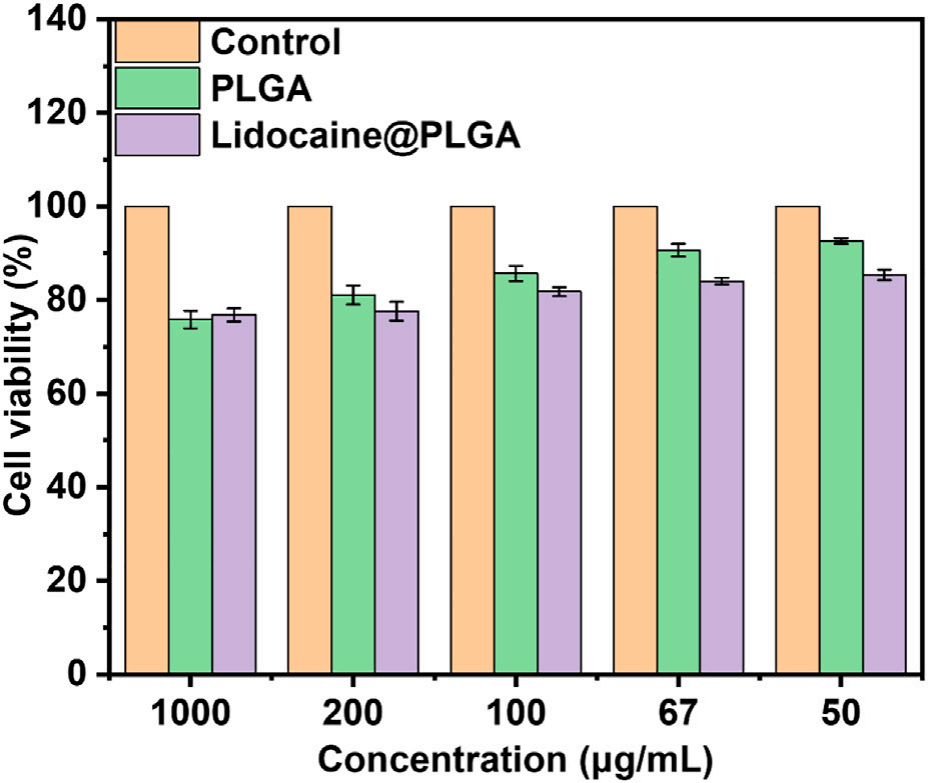
*In vitro* cytotoxicity of PLGA microcapsules with or without lidocaine laden.

Cytotoxicity was tested for the viability of different concentrations of Lidocaine@PLGA microcapsules co-incubated with L-929 cells for 24 h. Compared with the control group, the cell viability of the blank microcapsule group and the Lidocaine@PLGA microcapsules group reached 90.6% and 84.1% at the concentration of 67 μg/ml, respectively; at the concentration of 50 μg/ml, the cell viability of the blank microcapsule group and Lidocaine@PLGA microcapsules was 92.6% and 85.4% respectively, indicating that the drug-loaded microspheres had good biocompatibility, safety, and non-toxicity in solution. As the concentration increased, the cell viability of the blank microcapsule group and that of the Lidocaine@PLGA microcapsule group were gradually decreased to 81.1% and 77.7%, respectively, at a concentration of 200 μg/ml. However, further increase of the concentration into 1000 μg/ml, the cell viability could still maintain the 75.8% and 76.9%, respectively, as shown in Figure 6. The drug-loaded microcapsule group had similar biocompatibility to the blank microcapsule group, which not only suggested that the drug had good stability in the microcapsule but also demonstrated that a drug-loaded microcapsule with good biocompatibility can be expected for the treatment of sciatica, even in areas that require higher drug concentrations.

### Therapeutic effect of animal models

**FIGURE 7:**
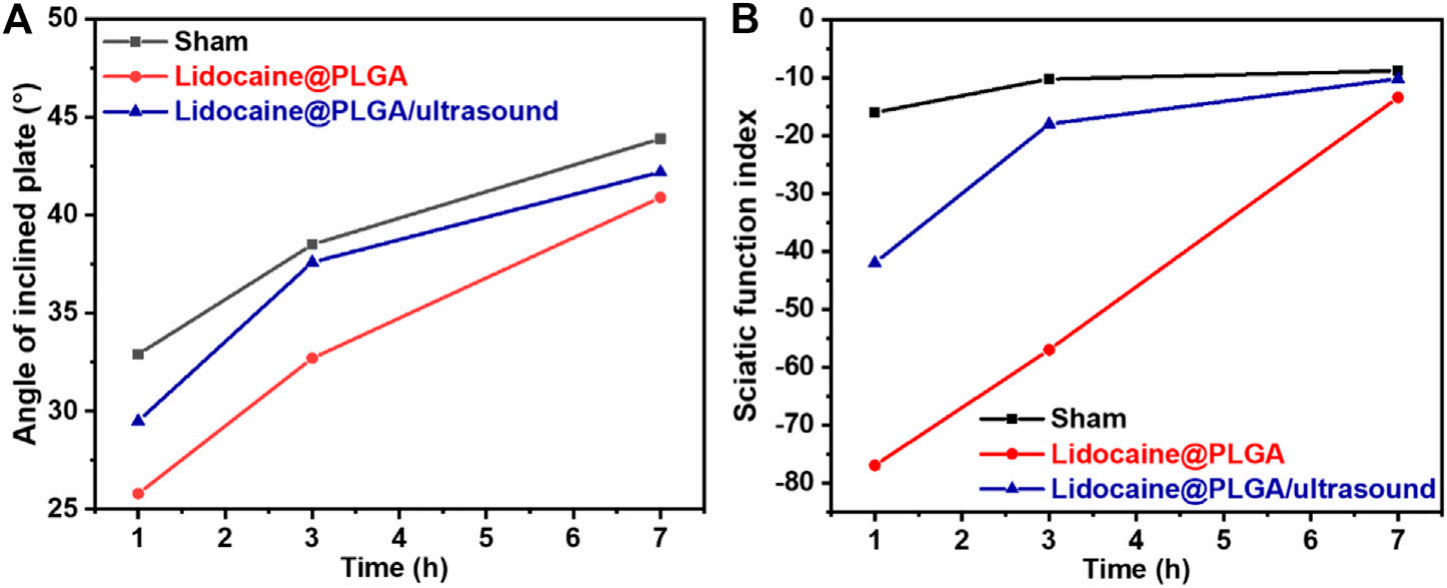
SD rats were tested for **(A)** sciatic nerve function index and **(B)** inclined plate experimental parameters after treatment at different times.

Each rat in the Lidocaine@PLGA group and Lidocaine@PLGA/ultrasound group was percutaneously injected into the sciatic nerve with 0.2 ml aqueous solution (equivalent to 1% lidocaine 6 mg), and the sciatic nerve function index measurement and oblique plate experiment were measured after injection for 1, 3, and 7 h, wherein Lidocaine@PLGA/ultrasound group was exposed to a 6 min of ultrasound intervention before each measurement. As shown in Figure 7, the sham group underwent measurements of sciatic nerve index and inclined plate experiments at the same time point. The results showed that at 1 or 3 h after the injection of Lidocaine@PLGA microcapsule, the sciatic nerve function index and inclined plate angle of rats after ultrasound intervention were significantly better than those of rats without ultrasound intervention, and the sciatic nerve function index and oblique plate angle of rats in the non-ultrasound intervention group were close to those in the ultrasound intervention group at 7 h. This may be because the earlier ultrasound had made a lot of lidocaine release from the microcapsules, and thus, the released drug concentration was not enough in the sciatic nerve in the late stage compared to that of the sustainable slow release of Lidocaine@PLGA microcapsule. In this case, the total drug concentration remained roughly the same after 7 h between these two groups. In other words, in the ultrasound intervention group, some of the remaining microcapsules underwent ultrasound intervention at 1 and 3 h, and the drug in the microcapsules within the 7 h may have been lower than that of the non-ultrasound intervention group. Likewise, the amount of drug release was lower than that of the non-ultrasound intervention group, producing the ultrasound intervention group and the rat-inclined plate experiment and sciatic nerve index results of the non-ultrasound intervention group. The sciatic nerve index of Lidocaine@PLGA/ultrasound group rats rose from −42 to −10.17 at 1 h and from 29.5 to 42.2 in the inclined plate experiment within 7 h after administration. In the Lidocaine@PLGA group, the sciatic nerve index increased from −77 to −13.4 within 7 h and the slanted plate experiment rose from 25.8 to 40.9. As for the sham group, the sciatic nerve index increased from −16 to −8.8 and the angle of the inclined plate increased from 32.9 to 43.9. It was noted that the rats in the sham group also showed sciatic nerve damage, which may be because in the early stage of the experiment, the rats did not adapt to the test environment and could not walk according to the usual gait, and some rats even appeared to jump and walk, thus resulting in incomplete footprints and errors in data collection.

## Conclusion

We developed an ultrasound-responsive Lidocaine@PLGA microcapsule by using a premixed membrane emulsification method. After optimization of ultrasound instrument and duration time, the Lidocaine@PLGA microcapsule can achieve a stimulus response even under simulated skin conditions. In addition, the *in vivo* results also showed that the Lidocaine@PLGA microcapsules could perform rapid ultrasound-responsive drug release and relieve the sciatica nerve pain under the rat model of sciatica. Importantly, the ultrasound-responsive drug release not only provided more chances for the rapidly increasing local drug concentration under ultrasonic conditions but also ensured long-term sustained release without outside intervention, demonstrating a true on-demand administration of drugs, which made up for the short-term control of the release rate of the sustained-release microcapsules in clinical applications. Using ultrasound as the trigger switch of the Lidocaine@PLGA microcapsule, the drug release of microcapsule can be feasibly adjusted, and the individualized diagnosis and treatment plan can be adopted according to the specific situation in clinical application.

## Data Availability

The raw data supporting the conclusion of this article will be made available by the authors, without undue reservation.
